# Proton and helium ion radiotherapy for meningioma tumors: a Monte Carlo-based treatment planning comparison

**DOI:** 10.1186/s13014-017-0944-3

**Published:** 2018-01-09

**Authors:** Thomas Tessonnier, Andrea Mairani, Wenjing Chen, Paola Sala, Francesco Cerutti, Alfredo Ferrari, Thomas Haberer, Jürgen Debus, Katia Parodi

**Affiliations:** 1 Department of Radiation Oncology, University Hospital Heidelberg, Heidelberg, Germany; 2 Heidelberg Ion Beam Therapy Center, Heidelberg, Germany; 3 Centro Nazionale di Adroterapia Oncologica, Pavia, Italy; 4 Department of Medical Physics, Ludwig-Maximilians-Universität München, Munich, Germany; 5 Istituto Nazionale di Fisica Nucleare, Sezione di Milano, Milan, Italy; 6 European Organization for Nuclear Research, CERN, Geneva, Switzerland

**Keywords:** Helium ions, Protons, Meningioma, Monte Carlo, FLUKA, Treatment planning

## Abstract

**Background:**

Due to their favorable physical and biological properties, helium ion beams are increasingly considered a promising alternative to proton beams for radiation therapy. Hence, this work aims at comparing *in-silico* the treatment of brain and ocular meningiomas with protons and helium ions, using for the first time a dedicated Monte Carlo (MC) based treatment planning engine (MCTP) thoroughly validated both in terms of physical and biological models.

**Methods:**

Starting from clinical treatment plans of four patients undergoing proton therapy with a fixed relative biological effectiveness (RBE) of 1.1 and a fraction dose of 1.8 Gy(RBE), new treatment plans were optimized with MCTP for both protons (with variable and fixed RBE) and helium ions (with variable RBE) under the same constraints derived from the initial clinical plans. The resulting dose distributions were dosimetrically compared in terms of dose volume histograms (DVH) parameters for the planning target volume (PTV) and the organs at risk (OARs), as well as dose difference maps.

**Results:**

In most of the cases helium ion plans provided a similar PTV coverage as protons with a consistent trend of superior OAR sparing. The latter finding was attributed to the ability of helium ions to offer sharper distal and lateral dose fall-offs, as well as a more favorable differential RBE variation in target and normal tissue.

**Conclusions:**

Although more studies are needed to investigate the clinical potential of helium ions for different tumour entities, the results of this work based on an experimentally validated MC engine support the promise of this modality with state-of-the-art pencil beam scanning delivery, especially in case of tumours growing in close proximity of multiple OARs such as meningiomas.

**Electronic supplementary material:**

The online version of this article (10.1186/s13014-017-0944-3) contains supplementary material, which is available to authorized users.

## Background

In comparison to conventional photon radiation, ion beams offer favorable physical and biological properties, which may enable maximizing the dose delivered to the tumour volume, while reducing the dose to sensitive organs at risk (OAR) and the integral dose to the patient. To date, about 70 ion therapy facilities worldwide treat patients with ion beams, predominantly protons and, to a lower extent, carbon ions. However, synchrotron-based multi-source facilities such as the Heidelberg Ion Beam Therapy Center (HIT [1]) can offer the possibility to produce and accelerate to therapeutic energies additional ion species such as helium and oxygen, thus making an initial evaluation of their treatment effectiveness via *in-silico* treatment planning studies highly desirable. In particular, compared to the first clinical experience of helium ions with passively scattered beam delivery at the Lawrence Berkeley Laboratory [2, 3], modern facilities such as HIT can provide fully active pencil beam scanning delivery.

For tumour entities, which do not necessarily require an elevated linear energy transfer (LET) and associated relative biological effectiveness (RBE) of heavy ions, helium ions delivered with state-of-the-art techniques have the potential to improve clinical outcome in comparison to the more broadly deployed proton beams. In terms of physical interactions properties, the reduced range and lateral straggling of helium ions compared to protons [4, 5] can result in superior healthy tissue sparing and improved dose-to-target conformity. In terms of radiobiology, helium ions exhibit a higher LET and therefore a higher RBE than protons, as investigated in [6], as well as a smaller oxygen enhancement ratio (OER). This trend continues for heavier ions such as carbon that can reach even higher LET values and correspondingly higher RBE values, besides offering a reduced distal and lateral straggling in comparison to helium ions. Nevertheless, helium ions exhibit a favorably reduced fragmentation tail after the Bragg peak compared to heavier ions, thereby delivering lower dose distal to the target [4]. Moreover, the variation in RBE between tumor and normal tissue – the so called differential effect – is reduced for helium ions compared to heavier ions. Thus the lower range of RBE values makes biological dose predictions of helium ions less prone to uncertainties, particularly in normal tissue. Hence, all these considerations make helium ions a promising treatment option offering possibilities of dose escalation compared to current clinical practice, owing to their reduced penumbra compared to protons and their lower dose fragmentation tail compared to heavier ions. In particular, helium ion treatments could effectively reduce dose to OARs located in proximity to the target, as well as reduce the integral dose delivered to the patient with the related risk of radiation-induced secondary cancer. Vernimmen et al. [7] noted the particular interest of proton beam therapy for complex brain tumour entities, such as meningiomas. For these indications, helium ions hold a great potential to improve the therapeutic gain even further with respect to proton therapy.

Due to the growing interest in helium ions as an alternative to proton beams, recent works investigated their advantages in *in-silico* treatment planning studies [8–10], however relying on research platforms not validated against experimental dosimetric data. To overcome these limitations, we first performed a thorough dosimetric characterization of helium ion beams in water and air [4], as well as an in-depth validation of the Monte Carlo (MC) code FLUKA [11] and the related research tool for MC-based treatment planning [5, 12, 13]. In a next step, our FLUKA-based calculation framework was combined with a validated phenomenological biological model, previously benchmarked against data for proton as well as helium ions, and capable to account for the entire mixed radiation field generated in nuclear interaction [14–16]. The use of MC throughout and LET-based RBE modelling is an advancement over previous publications [8–10]. This way, we could develop the first thoroughly validated (both in terms of physics and biological modeling) treatment planning research platform, which has been used in this work to perform a treatment plan comparison of four different brain and ocular meningioma cases, using protons and helium ions.

## Methods

### Meningioma patient cases

Four meningioma patient cases treated at HIT with protons, assuming a constant RBE of 1.1, were used in this study. Details of each selected case are presented in Table 1. In particular, our selection included:

- three cases of brain meningiomas irradiated with two beams (patients A-C);
- one case of optical meningioma at shallow depth, irradiated with a single beam (patient D).

**Table 1:** Proton plans characteristics for the considered patient cases, as extracted from the TPS. The dose per fraction, number of fractions and number of beams are reported. The table also provides information on the angles between beams and the OARs taken into account for the optimization

Patient	Planned dose/fraction [Gy(RBE)]	Number of fractions	Number of beams	Angles between beams [°]	Critical organ at risks
A	1,8	29	2	180	*Temporal lobes (left/right)*
					*Brain stem*
					*Optical system*
B	1,8	30	2	75	*Brain stem*
					*Chiasma*
					*Optic nerves (left/right)*
					*Intra-auricular nerve (right)*
C	1,8	30	2	32	*Brain stem*
					*Chiasma*
					*Optic nerve (right)*
D	1,8	28	1	\	*Optic nerve (left)*
					*Eye (left)*

**Fig. 1:**
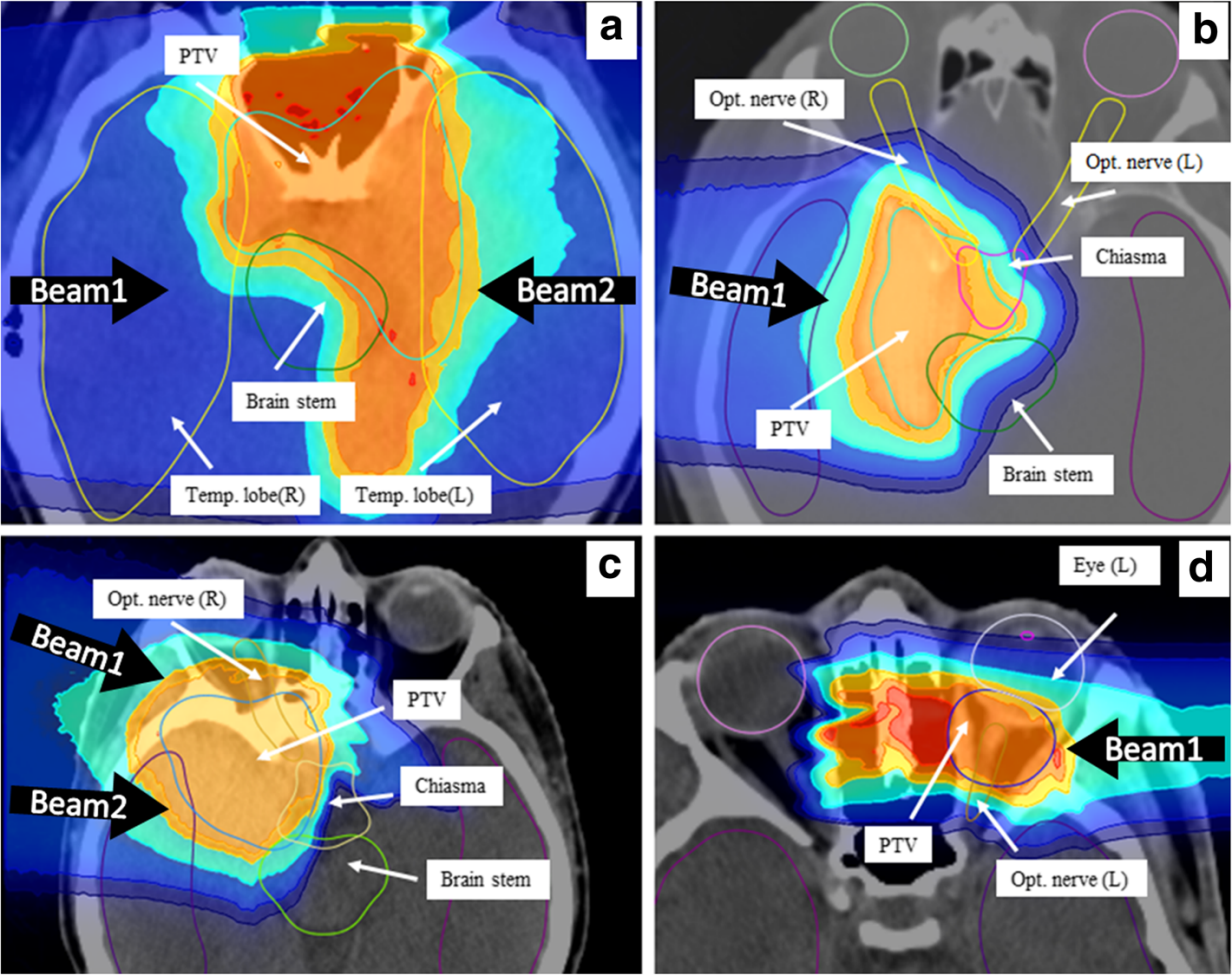
The four panels (**a**-**d**) display the complex relation between the contoured PTV and OARs for the four considered patient cases on the X-ray computed tomography axial images (gray scale), with overlaid dose distributions (color wash) for proton plans of fixed RBE calculated with MCTP. Second beam for patient B (panel b) is following the head-feet axis

The spatial locations of the critical OARs (cf. Table 1) relative to the planning target volume (PTV) are displayed in Fig. 1. The original proton plans from the commercial Treatment Planning System (TPS, SyngoPT, Siemens) were re-optimized with the FLUKA-based Monte-Carlo treatment planning tool (MCTP [5, 12, 13]) for both protons and helium ions, using a variable RBE model for both ions, as well as a fixed RBE value of 1.1 for protons.

RBE of protons in clinical practice is defined at 1.1 [17], since, as explained in [18], there are no sufficient in-vivo data to support a variable RBE approach. However some radiobiological studies especially in vitro with given cell lines have shown a rise in RBE with the increase of LET [16, 19], justifying the implementation of such variable model in the MCTP.

For biological dose calculations using variable RBE schemes, the α_x_ and β_x_ terms from the linear quadratic equation [20] for photon irradiation need to be defined for each tissue. However, with the biological model of [15, 16] selected in this work, the α_x_/β_x_ ratio is sufficient to perform biological calculation. Two regions of interest (ROIs) were distinguished for this study: the target (PTV) and the OAR. For the non-tumorous regions, a tissue type with an α_x_/β_x_ ratio of 2.0 Gy [21] was chosen. For the PTV, the tissue-type representing meningioma was used, with an α_x_/β_x_ ratio of 3.7 Gy, as recommended in [22].

### Optimization methods and dose calculation

The MCTP tool, presented in [13] for protons and in [12] for carbon ions, was extended to helium ion beams in this work. For treatment planning, physical and biological optimization matrices were calculated from the initial TPS proton plans (i.e., same couch positions, gantry angles, depths of the energy layers) using the FLUKA code (*development version 2016* [23–25]) in the voxelized geometry of the patient computed tomography (CT) data with a voxel size of 1 × 1 × 3 mm^3^. From these plans and matrices, a first optimization for protons with a fixed RBE of 1.1 was performed to identify the best constraints on the ROIs achieving similar or better dose volume histograms (DVH) than the original plan from the analytical TPS. This approach has two main advantages: (1) it makes the DVH results readily comparable to those of the clinical TPS plans and (2) the biological dose is not subject to variations when changing tissue type or dose per fraction.

Optimizations with the identified constraints were then performed for protons and helium ions taking into account a phenomenological variable RBE approach [14–16]. The ripple filter [26] was used for helium ions to avoid possible target dose inhomogeneity, as seen for shallow target depths in [5].

For the PTV, constraints to achieve a biological dose of 1.8 Gy(RBE) per fraction were initially applied, as well as a constraint on the maximum dose, where 5% or more of the volume (D5_PTV_) should not receive more than 107% of the planned dose. For the OAR, a constraint on D5_OAR_ was set, close to the D5_OAR_ extracted from the DVH of the TPS plan. The D5 was chosen instead of D2 to define the maximum dose, since it was shown in [27] to be less prone to statistical variation in MC simulations. Additionally to the constraint on the OAR maximum dose, low dose constraints were applied with smaller optimization weights, to reduce as much as possible the volume of OAR receiving low dose, and thereby the integral dose by increasing the dose gradient between PTV and surrounding normal tissue. In fact, while multiple DVH constraints on dose and volumes can be directly applied in the clinical TPS, this feature is currently under development for the MCTP tool.

After plan optimization, a final dose calculation was performed for each case (i.e., protons with fixed or variable RBE, helium ions with variable RBE) using 50 × 10^6^ primary histories in 50 independent runs, at 1 × 1 × 3 mm^3^ resolution. Dose distribution and DVH analysis for plan comparisons were calculated and plotted with an in-house software [28]. All considered parameters were calculated for the full treatment dose, i.e., *planned target dose* multiplied by the *number of fractions* (cf. Table 1). In particular, the following DVH parameters were extracted for plan evaluation of each ROI: D5, D10, D20, D33, D50, D66, D75, D95.

## Results

### MCTP optimized plans in comparison to TPS plans for protons at fixed RBE

The DVH results of proton optimization with fixed RBE for the MCTP compared to the TPS are shown in Table 2, representing the baseline dose difference between dose volume parameters of the MCTP plans against the TPS plans. For the PTV of the four patients, the D5_PTV_ value obtained with MCTP was found higher by a maximum of about 2 Gy(RBE) compared to TPS. However, overall a similar coverage of the target was achieved, with on average less than 1.5 Gy(RBE) difference for all extracted DVH parameters. The maximum deviation of 3 Gy(RBE) was found for the D95_PTV_ of patient A.

**Table 2:** DVH parameters difference, in Gy(RBE), between the MCTP optimized proton plans with fixed RBE and the original TPS plans for all the investigated ROIs of the four patients

		DVH analysis: H (MCTPS, RBE = 1.1) - H (TPS, RBE = 1.1)							
Patient	ROI	D5	D10	D20	D33	D50	D66	D75	D95
A	**PTV**	2,0	1,7	1,2	0,9	0,6	0,0	0,0	-3,2
	*Temporal lobe left*	-4,1	−2,3	0,0	0,6	0,9	0,6	0,6	0,0
	*Temporal lobe right*	−6,7	−3,8	−4,4	−1,7	−1,5	−1,7	−2,0	−0,3
	*Brain stem*	−0,9	−2,0	−1,7	−1,5	−0,6	0,0	−0,3	0,0
	*Optical system*	−3,5	−4,9	−7,5	−9,9	−8,1	−5,2	−1,2	0,0
B	**PTV**	1,5	0,9	0,3	0,6	0,3	0,3	0,3	−1,8
	*Optic nerve left*	−0,9	−1,5	−0,3	0,3	−0,3	0,0	0,0	−0,3
	*Chiasma*	1,5	1,2	0,3	−1,8	−2,1	0,6	2,1	2,1
	*Optic nerve right*	2,1	1,8	0,9	−0,9	−0,6	0,9	0,6	0,0
	*Brain stem*	−3,0	−5,1	−9,0	−5,4	1,5	3,6	2,7	0,9
C	**PTV**	0,6	0,6	0,0	0,3	0,3	0,6	0,6	−0,9
	*Optic nerve (right)*	1,2	1,5	1,2	0,6	0,9	0,9	0,9	1,8
	*Chiasma*	0,3	0,0	−2,7	−9,0	−3,9	−5,4	−0,3	−0,3
	*Brain stem*	−9,6	−7,2	−1,5	0,9	0,3	0,0	0,0	−0,3
D	**PTV**	2,0	1,7	1,4	0,6	0,3	0,3	0,3	1,1
	*Eye (left)*	1,4	2,2	3,4	3,6	3,6	3,1	2,8	1,4
	*Optic nerve (left)*	−3,1	−2,5	−2,5	−0,8	2,8	3,4	2,8	0,8

Regarding the OARs, a relatively good agreement in terms of the maximum dose was obtained, with less than 2.1 Gy(RBE) difference in the D5_OAR_. For the other OAR DVH parameters, the difference between MCTP and TPS was below 2 Gy(RBE), except for patient D and the brainstem of patient B. For patient D and the brainstem of patient B, MCTP was giving higher dose on average to the OAR, up to 3.6 Gy(RBE) for the left eye of patient D. On the other hand, the MCTP was able to reduce the dose up to 10 Gy(RBE) in regions such as the optical system of patient A. For this latter patient, all OARs indicated a reduced dose with MCTP compared to the TPS plan. For patient C, D5_brainstem_ of brainstem was reduced by 9.6 Gy(RBE) with MCTP, and the D33_chiasma_ of chiasma was about 9 Gy(RBE) lower. These results are acceptable and demonstrate the treatment planning capabilities of MCTP, providing improved results than analytical TPS where possible, as also seen in [13]. Regarding patient D, the higher dose observed in the OAR can be attributed to the difference in beam modeling at shallow depth between TPS and MCTP. The TPS assumes a beam spread smaller than the experimentally validated one of MCTP, as seen in [29] for a patient suffering from arteriovenous malformation (AVM), and in agreement with the observations of [30] reporting similar shortcomings for the same TPS system. Due to this underestimation of the beam lateral size, the TPS model predicts lower dose to OARs at shallow depth. In contrast, the MCTP predicts a larger beam broadening than TPS, consistently with experimental data [29], and thus enhances the weight of the beams delivering dose in the middle of PTV to provide a good PTV coverage, while trying to fulfill OAR constraints. Although larger D5_PTV_ values can be found in the other patient cases, they are still respecting the limits of 107% set in the constraints. Compared to analytical TPS systems performing calculation in water of variable depth, MC dose predictions generally exhibit higher D5_PTV_ values as well as lowered D95_PTV_ values, due to the more realistic beam transport in heterogeneous materials.

### Comparison of MCTP optimized plans for protons and helium ions

**Fig. 2:**
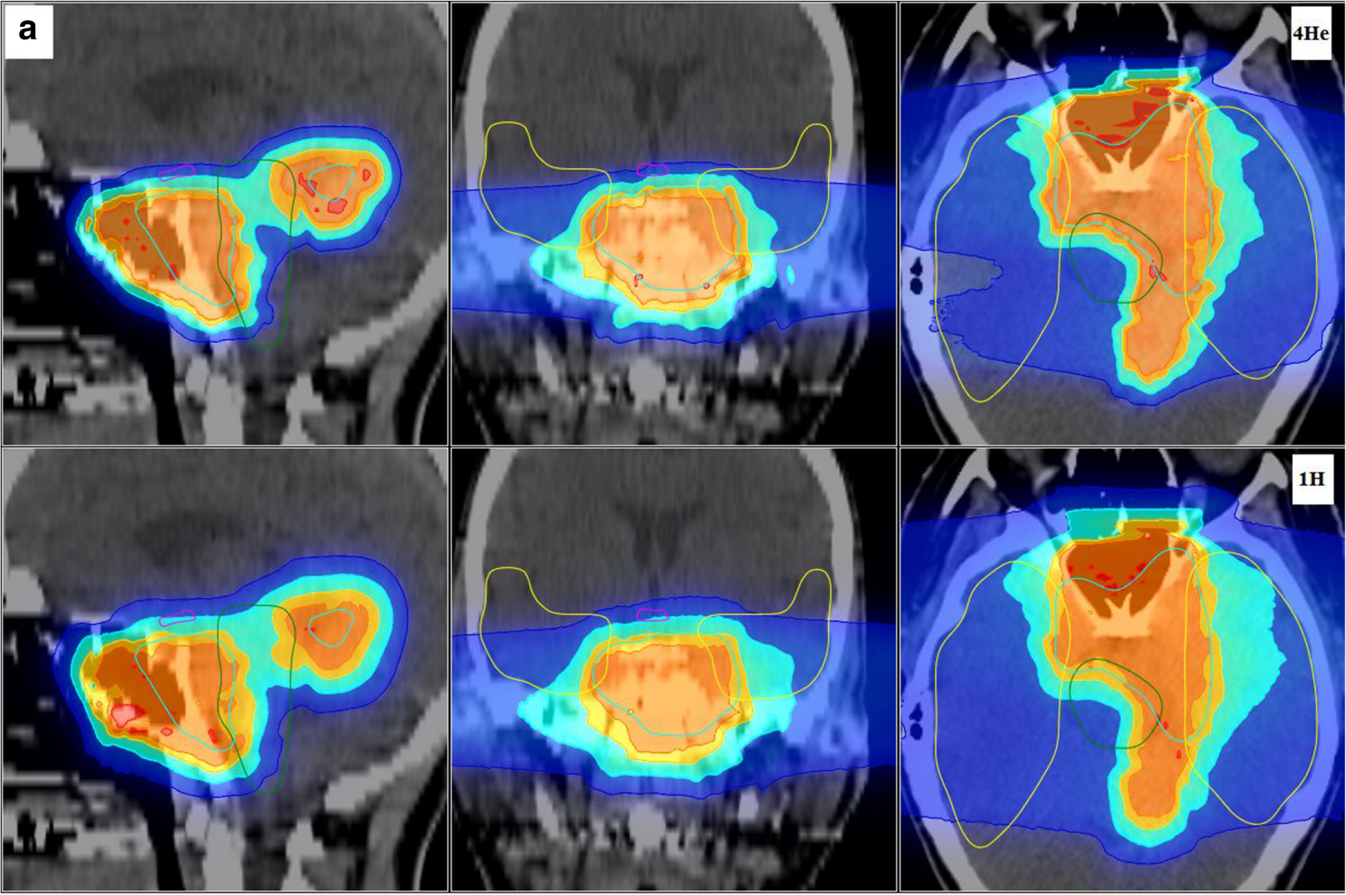
Planned dose distributions superimposed on the gray scale X-ray CT images are shown for patient A, featuring in the top panels helium ions (^4^He) and in the bottom panels protons (^1^H) for the sagittal (left), coronal (middle) and axial (right) slices

**Fig. 3:**
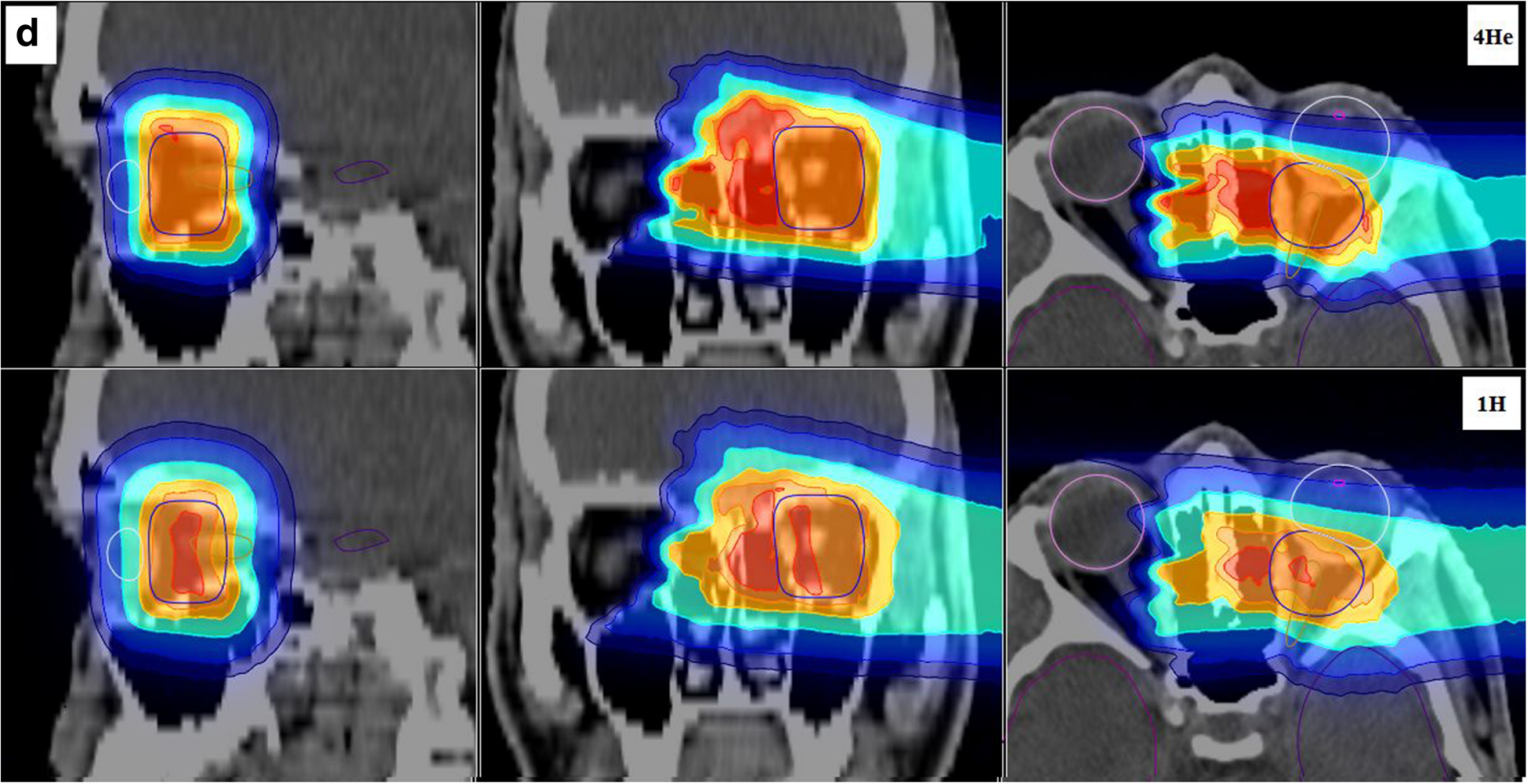
Planned dose distributions superimposed on the gray scale X-ray CT images are shown for patient D, featuring in the top panels helium ions (^4^He) and in the bottom panels protons (^1^H) for the sagittal (left), coronal (middle) and axial (right) slices

**Fig. 4:**
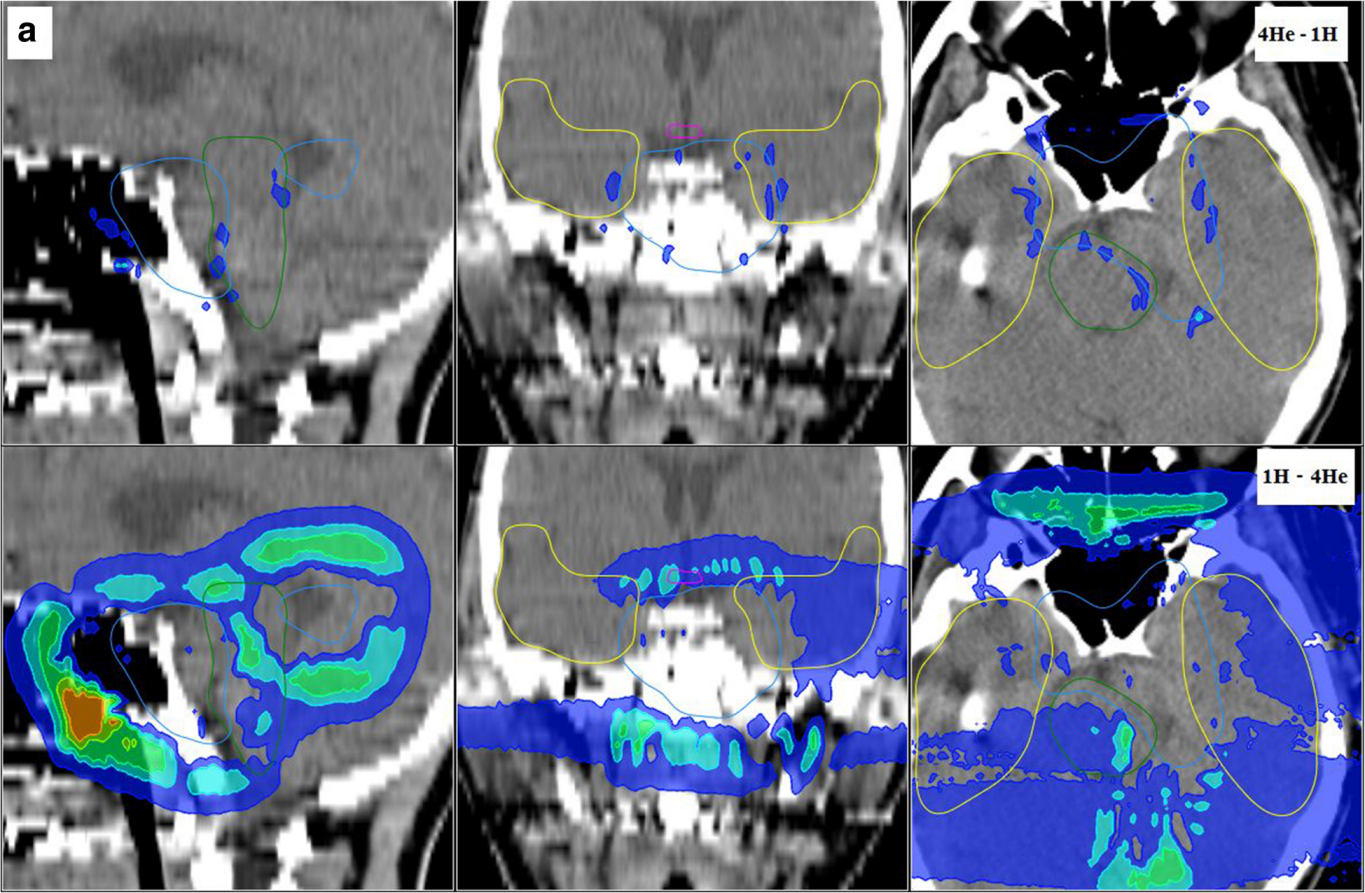
Dose difference between the helium ions and proton dose predictions for patient A: the top panel displays the overdosage due to helium ions (i.e., the difference of ^4^He dose minus the proton dose), while the bottom panel shows the overdosage due to protons (i.e., ^1^H-^4^He). Colors represent the dose >3, >6, >9, >12, >15 and >18 Gy(RBE) (from blue to red), for a 54Gy(RBE) total treatment dose

**Fig. 5:**
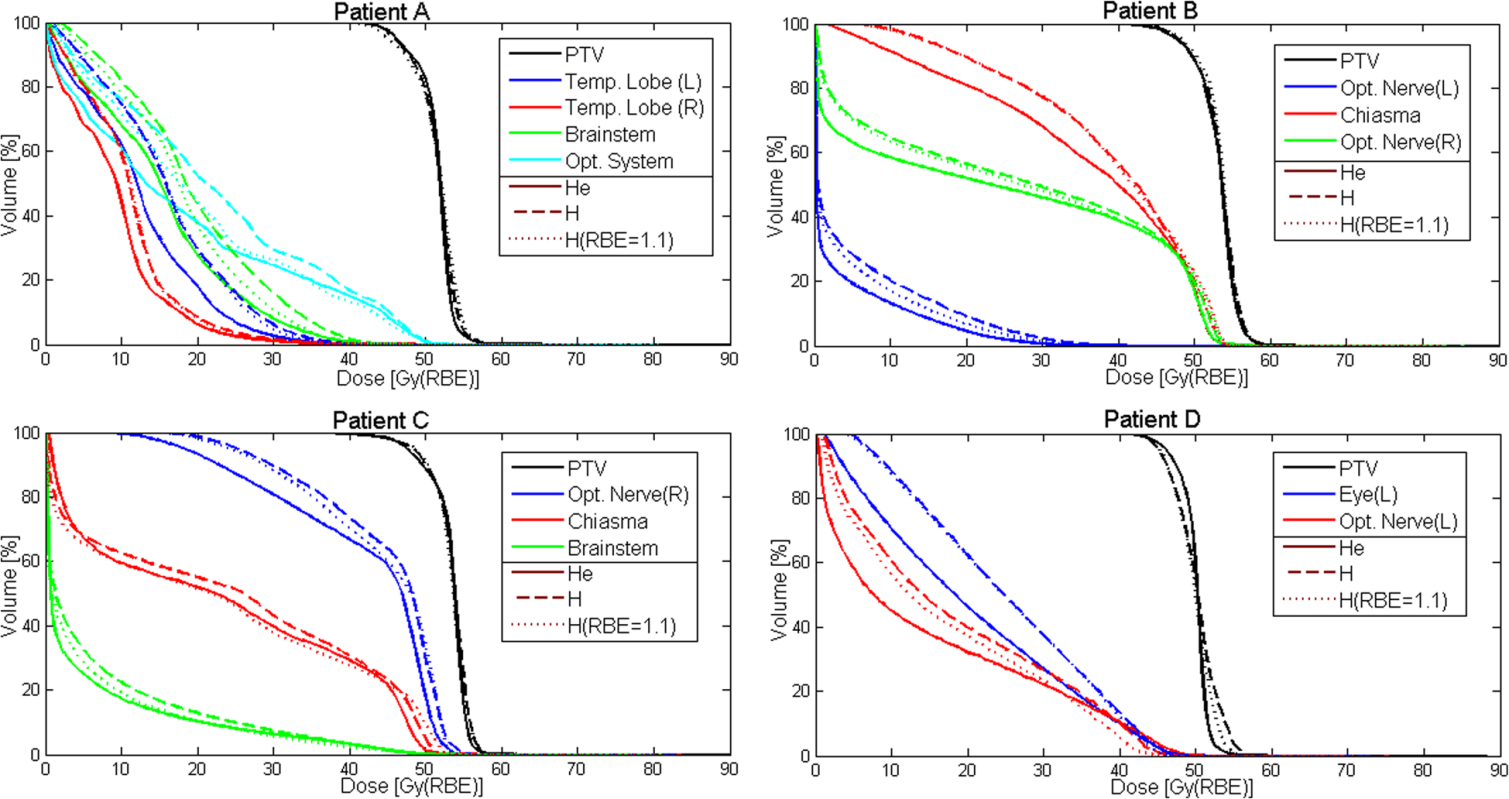
DVH for the four considered meningioma cases. The different ROIs investigated are displayed. Protons are shown for both fixed (dotted lines) and variable RBE (dashed lines). Helium ions are displayed with solid lines

The dose distributions obtained with a variable RBE scheme are exemplarily shown for patient A and D in Figs 2 and 3, respectively, with the helium ion dose prediction on top and the one for protons on the bottom. The treatment plans for the other patients are reported in the Additional file 1. The chosen dose display uses a color-wash system showing selected dose level set to >10%, >20%, >50%, >80%, >95% and >107% of the prescribed dose. In Fig. 4 the difference between the planned dose distributions are shown, with the top panel showing the helium ions dose minus the protons one, while vice versa in the bottom panel, with a color-wash system showing the dose differences >3, >6, >9, >12, >15 and >18 Gy(RBE). It can be seen that the 10%, 20% and 50% dose level regions are broader for protons than for helium ions, as best visualized in the dose difference maps. The dose gradients outside the target are sharper for helium ions than for protons in all directions, as expected. In Fig. 2 for patient A, some hotspots can be observed for helium ions outside of the target volume or near the vicinity of the PTV. In addition to the shallow dose gradient found for protons for patient D, doses above 107% exist within the PTV for the proton patient D plan (cf. also Fig. 5).

DVH plots for the different ROIs are displayed in Fig. 5. Proton results are shown for both fixed (dotted lines) and variable RBE (dashed lines). Helium ions are displayed with solid lines. Tables 3 and 4 quantify the differences between DVH parameters extracted from helium ion and proton plans, the latter with either a fixed or variable RBE. As also visible in Fig. 5, the PTV coverage reported in Tables 3 and 4 was found similar for all cases except patient D, where helium ions afford better coverage, with a sharper PTV DVH. For this patient D, the D5_PTV_ is larger with protons by about 2 Gy(RBE), due to the hot spots previously mentioned. For most of the dose volume parameters in OARs, the dose is reduced with helium ions compared to protons with a fixed RBE (Table 3), with dose sparing up to 7.5 Gy(RBE) for D95_chiasma_ of Patient B. Only the D5_brainstem_ of patient C and D5_Opt.Nerve_/ D10_Opt.Nerve_ of patient D present higher doses above 2 Gy(RBE) for helium ions, with values of about 3 Gy(RBE) and 3.9/2.5 Gy(RBE), respectively. When comparing helium ions to protons with variable RBE, the helium ion dose in OARs is reduced up to 7.8 Gy(RBE) for D50_Opt.System_ of Patient A (Table 4). Only the D5_Opt.Nerve_ of patient D is exhibiting higher dose with helium ions by about 1.7 Gy(RBE). Proton plans optimized with variable RBE show in the OARs a DVH slightly shifted toward higher dose, about 1–2 Gy(RBE), compared to the plans optimized and calculated with fixed RBE value. This trend can be particularly seen for small volumes close to the PTV, for example leading to higher D5/D10 values.

**Table 3:** DVH parameters difference, in Gy(RBE), between the MCTP optimized helium plans with variable RBE (var. RBE) and proton plans with fixed RBE for all the investigated ROIs of the four patients

		DVH analysis: He (var. RBE) - H (RBE = 1.1)							
Patient	ROI	D5	D10	D20	D33	D50	D66	D75	D95
A	**PTV**	−0,9	−1,2	−0,9	−0,6	−0,3	0,6	0,9	1,2
	*Temporal lobe left*	−2,9	−3,8	−3,8	−4,1	−4,1	−3,5	−3,5	−1,7
	*Temporal lobe right*	−1,2	−1,2	−1,5	−1,5	−1,7	−2,9	−2,9	−0,9
	*Brain stem*	−1,5	−1,5	−2,3	−2,9	−2,3	−3,5	−3,8	−2,0
	*Optical system*	0,6	1,2	0,3	−2,0	−4,4	−4,6	−4,4	−1,7
B	**PTV**	−0,3	−0,3	0,3	−0,6	0,0	−0,3	−0,9	−0,6
	*Optic nerve left*	−3,3	−3,0	−3,6	−1,5	0,0	0,0	−0,3	0,0
	*Chiasma*	−1,2	−1,2	−0,9	−0,9	−2,1	−5,1	−6,3	−7,5
	*Optic nerve right*	−1,2	−1,2	−0,9	0,3	−4,2	−4,8	−2,1	−0,3
	*Brain stem*	−0,3	0,3	0,3	−3,0	−3,9	−3,3	−2,7	−0,6
C	**PTV**	−0,6	−0,6	0,0	0,0	0,6	0,0	0,3	−0,6
	*Optic nerve (right)*	−0,6	−1,2	−1,5	−1,5	−1,2	−1,5	−3,3	−6,0
	*Chiasma*	−2,4	−2,1	−0,6	2,1	0,9	0,9	1,5	0,9
	*Brain stem*	3,0	0,0	−1,2	−1,5	0,0	0,0	0,0	0,0
D	**PTV**	−1,7	−1,1	−0,8	0,0	0,6	0,8	1,4	0,6
	*Eye (left)*	0,6	−1,1	−3,1	−5,0	−6,4	−6,4	−6,4	−4,5
	*Optic nerve (left)*	3,9	2,5	0,0	−3,6	−4,8	−3,6	−2,8	−0,8

**Table 4:** DVH parameters difference, in Gy(RBE), between the MCTP optimized helium plans and proton plans with variable RBE (var. RBE) for all the investigated ROIs of the four patients

		DVH analysis: He (var. RBE) - H (var. RBE)							
Patient	ROI	D5	D10	D20	D33	D50	D66	D75	D95
A	**PTV**	−0,6	−0,9	−0,6	−0,3	0,0	0,3	0,6	0,6
	*Temporal lobe left*	−4,4	−4,6	−4,6	−4,1	−3,8	−3,5	−3,5	−2,0
	*Temporal lobe right*	−2,3	−1,5	−1,5	−1,5	−1,5	−2,6	−2,9	−0,9
	*Brain stem*	−3,8	−4,3	−5,2	−4,6	−3,5	−4,4	−5,2	−3,2
	*Optical system*	−0,6	−1,2	−3,2	−5,8	−7,8	−7,3	−5,8	−1,7
B	**PTV**	−0,3	−0,6	0,0	−0,3	0,0	0,0	0,0	−0,6
	*Optic nerve left*	−6,0	−6,0	−5,7	−2,4	−0,3	0,0	−0,3	0,0
	*Chiasma*	−0,9	−0,9	0,0	−0,3	−2,4	−5,1	−6,3	−7,8
	*Optic nerve right*	−0,6	−0,6	−0,6	−0,9	−6,0	−5,7	−2,1	−0,3
	*Brain stem*	0,3	0,0	−2,1	−5,1	−6,0	−5,1	−4,2	−1,5
C	**PTV**	−0,6	−0,6	0,0	−0,3	0,0	0,0	0,3	−0,6
	*Optic nerve (right)*	−0,9	−0,9	−1,2	−1,2	−1,5	−3,6	−5,4	−6,9
	*Chiasma*	−1,2	−1,2	−0,9	−1,5	−3,3	−0,9	0,9	0,9
	*Brain stem*	−0,9	−4,2	−3,9	−2,7	−0,3	0,0	0,0	0,0
D	**PTV**	−2,5	−2,0	−1,1	−0,6	0,0	0,8	1,4	0,8
	*Eye (left)*	−0,3	−1,7	−3,4	−5,0	−6,4	−6,7	−7,0	−5,0
	*Optic nerve (left)*	1,7	0,3	−2,8	−5,9	−6,4	−5,0	−3,9	−1,4

For patient D, as previously mentioned, the PTV coverage was found better with helium ions, with a larger D95_PTV_ value and a smaller D5_PTV_ value, which is an indicator of PTV dose homogeneity. This finding is ascribed to the small size of the target and the proximity of the OAR. In fact, due to the larger lateral and distal fall-off of proton beams, they cannot cover the target optimally without impacting the surrounding OARs. Thus, to provide a better coverage, the optimizer has to choose to allocate higher doses in the middle of PTV for protons, as seen in Fig. 3.

Compared to protons plans with variable RBE, helium ions provide better sparing of OAR in all cases, excluding the left optic nerve of patient D. Since in this latter case the left optic nerve is included in the PTV, higher doses in the OAR are to be expected due to the above mentioned better dose homogeneity within the PTV. In general, the OAR DVH curves of helium ions are shifted toward lower doses for two reasons. First, the reduced lateral straggling positively impacts the DVH with a reduced OAR dose, particularly when considering OARs volume above 1/3 of their total volume (Fig. 5). Second, a more favorable ratio is found between the RBE at high LET for α_x_/β_x_ of 3.7 (target) and the RBE at low LET for α_x_/β_x_ of 2 (for the surrounding non-tumour tissue). This RBE ratio between tumour and non-tumour tissue is in the order of 15–20% larger for helium ions for the studied cases, consistent with the model comparisons reported by [31], thus leading to less deposited physical dose than protons for the same prescribed biological dose to the target. Even when compared to proton irradiation with fixed RBE of 1.1, helium ions still present the advantage of superior OAR dose sparing, since protons with fixed and variable RBE showed similar trends. The largest differences between protons with variable and fixed RBE can be seen for OARs at the end of the beam range, where LET, and therefore RBE, is enhanced, ultimately leading to higher biological dose deposition. This latter case can be seen particularly for the chiasma of patient C in Fig. 5.

## Discussion

This work demonstrated the capabilities of the developed MCTP framework to provide with proper constraints sound treatment plans, comparable in quality to those obtained from the clinical TPS for protons with fixed RBE. The extension of MCTP to helium ions enabled performing an *in-silico* comparison with protons under realistic conditions, using for the first time a MC-based treatment planning platform thoroughly validated both in terms of physics and biological models for both ion species. The results of this study confirmed the anticipated advantages of helium ions over protons for meningioma cases at the considered fraction dose of 1.8 Gy(RBE), showing similar PTV coverage and better sparing of OAR for all beam configurations investigated, featuring two fields (from opposing to small angles) as well as single field. The benefits of both the physical advantages, in terms of improved lateral and distal straggling, and the more favorable biological properties, in terms of enhanced RBE in the target at the considered fraction dose, were particularly seen when using a variable RBE scheme for both ions.

Although not shown in this work, similar advantages were also observed when considering a hypofractionated delivery of helium ions at 3 Gy(RBE) dose per fraction, although the performance was found dependent on the tissue-specific α_x_/β_x_ ratio [32]. Additional investigations for the shallow target of patient D indicated that similar or even better planning results could be obtained when omitting the usage of the ripple filter, especially when introducing a tighter separation of energy layers corresponding to 1 mm Bragg peak spacing in water, instead of the typical separation of ~2 mm used by the TPS [32].

## Conclusion

Helium ions can be considered a promising treatment modality for low-grade meningiomas, where several critical structures to be spared are surrounding the tumour, and elevated RBE values are not necessarily needed, in contrast to more aggressive high-grade malignancies. Moreover, additional indications might benefit from the above-mentioned advantages, and will be further explored with the developed MCTP tool.

Additional investigations using tumor control probability and normal tissue complication probability models or robust planning, to take into account the planning and delivery uncertainty, could also be of interest for a future introduction of helium ion therapy in the clinical routine. Efforts for tighter conformation of target-dose with better sparing of normal tissue and OARs will also largely benefit from the possibility of in-vivo verification, where encouraging results of few millimeters localization accuracy have been recently reported with Positron-Emission-Tomography [33, 34] and prompt gamma imaging [35], especially for tumours located in the head.

## Additional file

Additional file 1: Additional comparison of planned dose distributions for protons and helium ions for patients B and C. (DOCX 2541 kb)

## Abbreviations

AVM: Arteriovenous malformation
CT: Compute tomography
DVH: Dose volume histogram
HIT: Heidelberg ion beam therapy center
LET: Linear energy transfer
MC: Monte Carlo
MCTP: Monte Carlo treatment planning system
OAR: Organ at risk
OER: Oxygen enhancement ratio
PTV: Planning target volume
RBE: Relative biological effectiveness
ROI: Region of interest
TPS: Treatment planning system
