# Supplementary material for: Proton and helium ion radiotherapy for meningioma tumors: a Monte Carlo-based treatment planning comparison
Source: Radiat Oncol. 2018 Jan 9;13:2. doi: 10.1186/s13014-017-0944-3 (PMC5759862; doi:10.1186/s13014-017-0944-3)
Supplement: Additional file 1: — Additional comparison of planned dose distributions for protons and helium ions for patients B and C. (DOCX 2541 kb) [file 13014_2017_944_MOESM1_ESM.docx]

**Appendix - Supplementary materials**


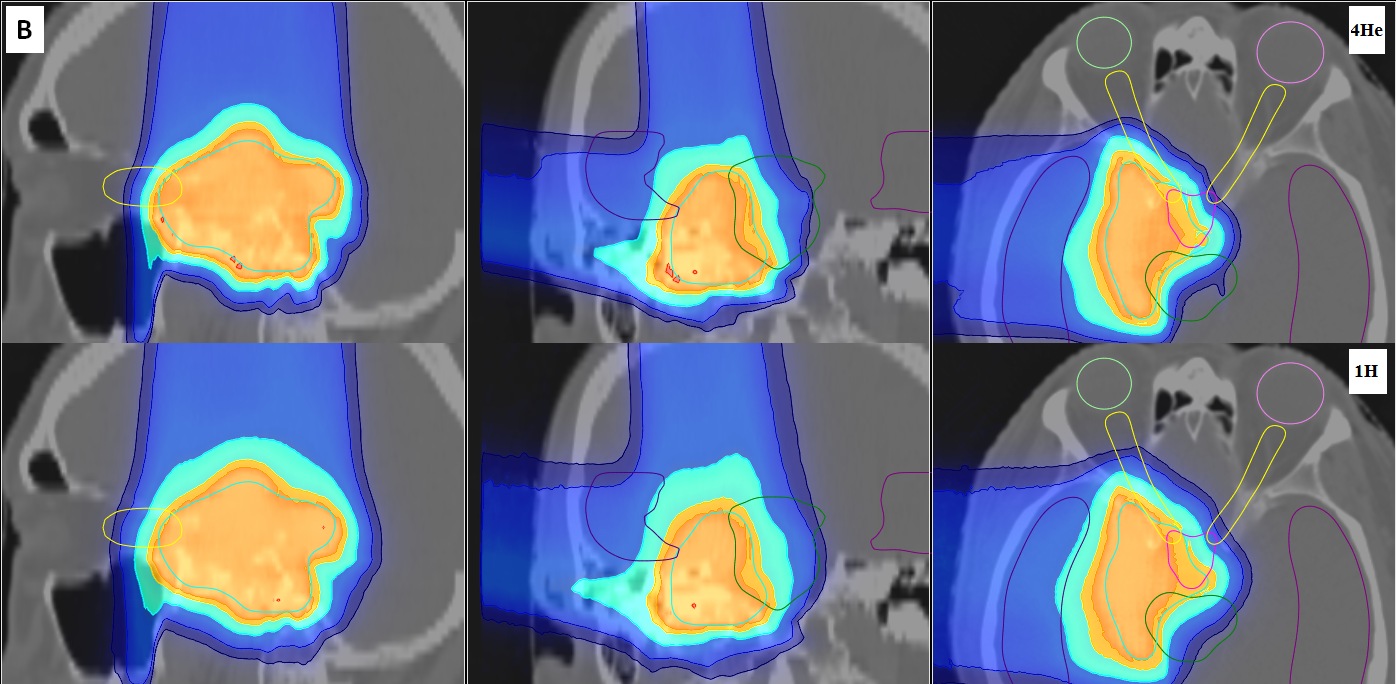


Appendix – Figure A: Planned dose distributions superimposed on the gray scale X-ray CT images are shown for patient B, featuring in the top panels helium ions (4He) and in the bottom panels protons (1H) for the sagittal (left), coronal (middle) and axial (right) slices.


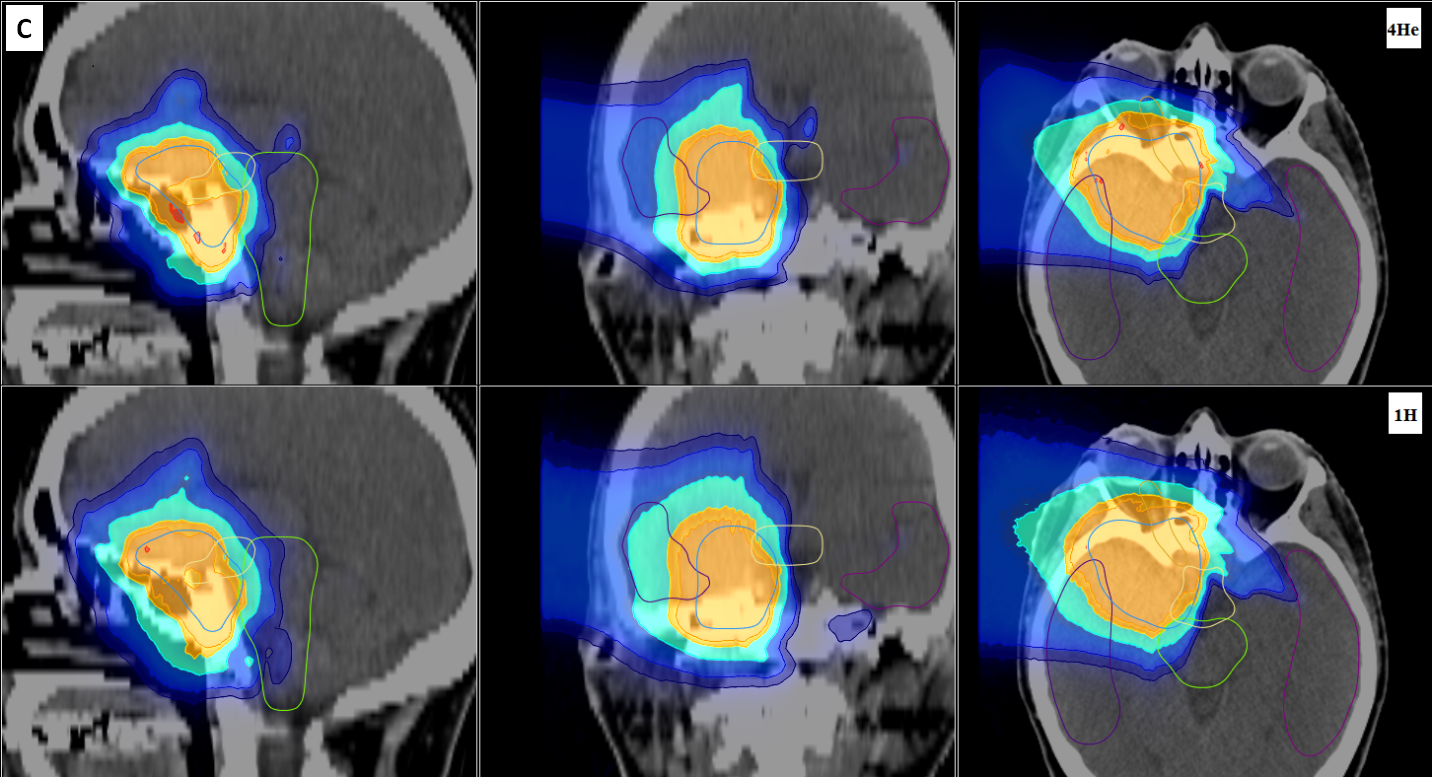


Appendix – Figure B: Planned dose distributions superimposed on the gray scale X-ray CT images are shown for patient C, featuring in the top panels helium ions (4He) and in the bottom panels protons (1H) for the sagittal (left), coronal (middle) and axial (right) slices.
